# 49 Characteristics of combined movement behaviour interventions in children and adolescents: A scoping review

**DOI:** 10.1093/eurpub/ckae114.047

**Published:** 2024-09-26

**Authors:** Ciaran Maloney

**Affiliations:** University Of East Anglia, Norwich, United Kingdom

## Abstract

Evidence suggests that targeting physical activity, sedentary behaviour, and sleep in combination can benefit health and academic outcomes in young people. This scoping review aimed to describe the extent, range, and nature of combined movement behaviour interventions and examine recruitment and effectiveness patterns in disadvantaged populations. The following electronic databases were searched: Web of Science, CINAHL, MEDLINE, and PsycINFO. Grey literature was identified through ProQuest Dissertations and Theses Global, Google Scholar, and the British Library EThOS. Included studies were randomised or quasi-experimental interventions that modified two or more movement behaviours with the goal of affecting health-, behavioural- or academic-related outcomes in children or adolescents. Peer-reviewed publications from scientific databases, master’s level dissertations, and doctoral theses from grey literature searches in the English language were included. The behaviour change technique taxonomy (BCT) and PROGRESS-Plus framework were used to map intervention characteristics. Twenty-eight studies met the inclusion criteria. Most studies were individual-level randomised controlled trials (43%), conducted in Europe (39%), and delivered in a school setting (75%). Physical activity and sedentary behaviour were the predominant behaviours that were modified (82%). The most commonly used BCTs included information about health consequences (68%) and social support [unspecified] (68%). All included studies focused on health-related outcome measures. Nine studies (32%) examined differential effects by PROGRESS-Plus subgroups. Future research should explore the value of movement behaviour interventions across the breadth of non-health-related outcomes and include a stronger focus on differential effectiveness in disadvantaged populations. No funding was received for conducting this study.

